# Influencing Factors of University Relocation on College Students’ Intention to Engage in Local Entrepreneurship and Employment

**DOI:** 10.3389/fpsyg.2021.750972

**Published:** 2021-12-13

**Authors:** Shihao Chen, Qianqian Zhang, Qun Zhao, Huiru Deng, Yu-Sheng Su

**Affiliations:** ^1^College of Science and Technology, Ningbo University, Ningbo, China; ^2^Department of Computer Science and Engineering, National Taiwan Ocean University, Keelung City, Taiwan

**Keywords:** family factors, local attraction, county education, employment, entrepreneurship

## Abstract

In modern society, the power of college students has been able to provide creative growth for the local economy, so the work situation of college students is closely related to the social dynamics. Colleges and universities are important places for talent cultivation and output. They are closely related to the cultivation of college students and the choice of employment and entrepreneurship of college students. Entrepreneurship and employment are interdependent. It is not enough to rely only on entrepreneurs to make enterprises stronger. In order to increase the creativity and environmental adaptability of enterprises, there need to be sufficient and excellent employees who are willing to work in the regions where enterprises are located. Therefore, enterprises need college students with innovation and creativity in their regions. In this study, graduates from a university in Zhejiang Province were selected as the subjects. Based on the Theory of Reasoned Action, literature analysis and interview method were combined to systematically construct a research model affecting college Students’ choice of entrepreneurship and employment. The questionnaire survey method and structural equation model (PLS-SEM) were used to test the hypothesis. A total of 798 valid questionnaires were collected. The results show that local attraction and family factors are the two most important factors affecting the entrepreneurship and employment of college students. Family factors have a significant positive impact on the attitude toward entrepreneurship and employment, subjective norms and intention to entrepreneurship and employment; local attraction also has a significant positive impact on the subjective norms, intention to entrepreneurship and employment, and attitude toward entrepreneurship and employment. The results of this study provide a reference for the decision-making of improving local attractiveness and promoting college Students’ employment in start-up enterprises under the background of higher education popularization.

## Introduction

University graduates are a country’s most precious source of talent. Attaching due importance to graduates, China issued “Opinions on Further Guiding and Encouraging College Graduates to Work County-Level Jobs” in 2017 to encourage graduates to work locally and pursue county-level infrastructure development. County education is a phenomenon in Zhejiang Province following the “new normal” of the economy and the rising prevalence of higher education. It is an essential part of Zhejiang Province’s third adjustment to its higher education deployment, after the establishment of universities and university towns in central cities following the Chinese economic reform (Hu, 2018). Universities are a crucial part of society. Likewise, college graduates are integral to social development. County college graduates seeking work and entrepreneurial opportunities locally and entrepreneurship is closely related to the allocation of human resources in county-level cities, economic development, and urban progress. However, most of China faces the problem of uneven university distribution and the loss of local talent. Therefore, this study explored the intention of college graduates to stay local for work and entrepreneurship, as well as the factors influencing the occupational intention of current students.

Exploring the occupational and entrepreneurial intentions of college graduates is particularly crucial in the context of harsh employment environments and the uneven regional distribution of talent. In 2020, the number of university graduates in China reached 8.74 million, a 0.4 million increase compared with the previous year. As a consequence of the economic recession and COVID-19 pandemic, graduate employment became increasingly difficult to secure. Every industry was affected to some degree, and the gap between supply and demand about college Students’ entrepreneurship and the employment market grew more pronounced. China’s job market prosperity index was substantially reduced, and new university graduates’ pursuit of entrepreneurship and employment appeared unpromising. Therefore, graduates’ entrepreneurship and employment become a major concern for all higher education stakeholders, including universities, the government, employers, and graduates themselves (Xie et al., 2017). In the future, regional enterprises in counties may face challenges in locating local and talented individuals to employ. Enabling university graduates to find employment within the local economy is the key step to revitalizing universities and the county as a whole. This would encourage university graduates to remain in the county and assist in the fundamental development and promotion of the local economy as well as the balance of county–city talent resource allocation. To attract talent to various counties and thereby promote industry growth and restructuring, the willingness of university students to remain in the county for entrepreneurship or employment purposes and relevant influential factors must first be assessed. Improvement measures can then be proposed, with the data serving as a valuable reference for future county employment development schemes. A review of the literature indicates that environmental factors are changing how career decisions are made (Callanan et al., 2017). Factors such as family education and parents’ educational background also affect the job location decisions of graduates (Rérat, 2014).

In research analyzing the factors influencing college graduates’ employment and entrepreneurship intentions, the theory of planned behavior (TPB) has been used as a theoretical basis for analysis, whereas studies have applied TPB to occupations (Wanberg et al., 2006; Van Hooft and De Jong, 2009) and job search (Van Hooft et al., 2004; Song et al., 2006; Saks et al., 2015; Su et al., 2020). The unique employment and entrepreneurship phenomenon in China is attributable to family culture, and young people’s schooling and employment problems are often resolved through family power. Abundant literature related to policies for university student employment and entrepreneurship have been published over the past few years. The structure of the employment and entrepreneurship policy system targeting college graduates is influenced by the diversity of policymakers. From the central to the local levels, regions have introduced policies on recruiting university students for employment and entrepreneurship. Therefore, the concept of county attractiveness was also included in this study. Further, the impact of the household registration system on the efficiency of labor allocation is also non-negligible. Overall, graduates’ job-seeking processes are influenced by local employment policies and family factors. An individual’s perceived behavioral control refers to their perception of the resources, opportunities, and capabilities they must possess to complete certain tasks (Ajzen and Madden, 1986; Bandura, 2012; Rodriguez-Gutierrez et al., 2020). However, in China, young people’s employment or entrepreneurship often relies more on the power of the family. In addition, the diversity of employment policy systems for Chinese university graduates as well as China’s household registration system greatly affect university Students’ employment. Thus, we determined that perceived behavioral control was not a crucial component of Chinese university Students’ job-seeking processes. Moreover, the Theory of Reasoned Action (TRA) has been widely applied in social psychology, a field that encompasses employment and entrepreneurship. Numerous studies have reported scholars’ application of the TRA to studies on employment and professional fields (Hooft et al., 2004; Van Hooft and De Jong, 2009; Wibowo and Indarti, 2020). Therefore, this study adopted the TRA to analyze university Students’ employment intentions. Numerous studies have explored the career decisions of college graduates (McDow and Zabrucky, 2015; Deer et al., 2018), but only a few have applied Theory of Reasoned Action to explore the factors affecting occupational decision making among college graduates at the county level (Joshi and Kuhn, 2011). Targeting the *status quo* of undergraduate education across Zhejiang Province, we applied TRA in empirical research to analyze graduates’ intention to stay local for work and entrepreneurship, as well as relevant impact factors.

In sum, this study applied TRA as its main theoretical framework and compared models with different demographic dimensions (i.e., family factors and country attractiveness). In doing so, the study investigated the effects of individual differences on graduates’ intentions to remain local. Accordingly, the following research questions are proposed:

1. Under the TRA model, do subjective norms and attitudes affect the intention of college graduates to stay local for work or entrepreneurship?
2. Do family factors affecting college graduates’ intentions to stay local for work or entrepreneurship vary?
3. Does the effect of county attractiveness on college graduates’ intentions to stay local for work or entrepreneurship vary?

Based on the TRA, relevant data were collected using both online and offline questionnaires. The research participants comprised students of the first local university in Ningbo City, China. Partial least squares structural equation model (PLS-SEM) was used to explore the factors affecting Chinese university Students’ willingness to remain in the county to pursue entrepreneurship and employment. The results of this study serve as a reference for future research into this crucial topic.

## Literature Review

### Theory of Reasoned Action

The TRA model, that is well and frequently utilized, has just lately been used in employment intention research (Joshi and Kuhn, 2011). Unlike other theories for vocational choice, TRA focuses on the effects of the social environment on occupational behavior and allows for a more detailed understanding of potential pull factors. Employment selection refers to an individual’s choice of being employed by a company or the government as well as setting up their own business. The pursuit of a profession (a type of behavior) can be predicted, with optimal results, by occupational intention. TRA involves two impact factors: (1) occupational attitude is defined by individuals’ overall evaluation of their occupational behavior, that is, motivating factors associated with employment choices, indicating the degree of effort individuals are willing to make to achieve employment goals; (2) subjective norms refer to one’s belief in significant others’ opinions and evaluations of one’s employment and entrepreneurship (Ajzen, 1991; Joshi and Kuhn, 2011; Conner, 2020; Mo et al., 2021). These factors indirectly affect occupational behavior through occupational intention, jointly determined by an individual’s attitude and perceived subjective norms about employment rather than directly affecting occupational behavior (Vincent et al., 1998; Joshi and Kuhn, 2011).

Occupational decision-making is often a major choice in a person’s life (Arnold et al., 2006). Joshi and Kuhn (2011) asserted that in most situations, the more positive one’s attitude toward a career, the stronger the desire to pursue it; however, the correlation is weak. Subjective norms represent how people perceive societal pressure to do (or not do) specific things. If an individual’s significant others deem the behavior positive (i.e., support the career choice) and the individual is encouraged to meet others’ expectations (i.e., are motivated to comply), they have greater motivation to pursue the career. If an individual’s significant others hold a negative attitude toward a career chosen by the individual and the individual wishes to meet their expectations, they tend not to pursue their chosen career. Fort et al. (2015) claimed that individuals exhibiting low levels of responsibility are unaffected by ethical rules and achievements, thus, they prioritize their personal attitudes when choosing employment. Van Hooft et al. (2004) reported that spending more time on job search leads to a higher probability of finding a job as well as a more favorable job offer. Therefore, unlike most studies on occupational decision-making, this research used TRA to gain a better understanding of the characteristics that influence Students’ future interest in jobs and entrepreneurship (Joshi and Kuhn, 2011). In addition, based on TRA, this study added two variables (i.e., county attractiveness and family factors) to explain occupational and entrepreneurship intentions.

### Local Attraction

Pull motivation represents the potential for a particular attraction to be consistent with someone’s motivational factors (Noela et al., 2017; Suni and Pesonen, 2017). Counties play pivotal roles in attracting university students through their policies on employment and entrepreneurship, salary, and benefits (Honeycutt and Rosen, 1997; Cable and Graham, 2000; Lievens and Highhouse, 2003; De Santis et al., 2021). Therefore, county attraction and motivational pulls are essentially the same. In this study, the concept of motivational pull was expanded to encompass county attractions and used to explore the employment and entrepreneurship intentions of university students. For employment and entrepreneurship in counties, county attractiveness is a crucial factor in the employment and entrepreneurship of college graduates. As noted by Noela et al. (2017) and Suni and Pesonen (2017), Students’ occupational motivation stems from personal needs that can be satisfied by being employed or starting their own business at their preferred location (Meng et al., 2008).

Therefore, occupational motivation is regarded as a crucial factor in explaining occupational behavior (Chang et al., 2014). Motivation is the main reason behind Students’ occupational behavior, and motivation is driven by the need for satisfaction (Suhartanto et al., 2018). Pull factors, which make the area where students live appear more attractive compared with other areas, play a key role in the employment and entrepreneurship of graduates locally. The main pull factors include employment policies and the cost of living. The cost of housing and the relationship between wages and real estate prices also affect people’s choice to live in a certain locality. A high cost of living is one reason why people are reluctant to live in certain areas. Concluded that real estate construction exerts a positive effect on population inflow. Other studies have reported that organizational attractiveness is influenced by applicants’ perceptions of the job and organizational characteristics such as salary, promotion opportunities, company location, career plan, and organizational structure (Turban and Keon, 1993; Honeycutt and Rosen, 1997; Cable and Graham, 2000; Lievens and Highhouse, 2003). Young people adapt to urban life for higher education and work (Brabyn and Jackson, 2019). Furthermore, income from work is a determinant of job satisfaction (De Santis et al., 2021). Therefore, enterprises in counties usually offer high salaries to retain graduates for local employment. In addition, the difficulty in the employment of college students often lies in unreasonable talent allocation. In cities, talents are highly concentrated, and entrepreneurship competition is fierce. Conversely, counties face a lack of employable talent. Counties must strategically use their regional advantages to attract college students wishing to start a business and utilize these Students’ entrepreneurial pursuits to trigger local college student employment (Qu et al., 2014). In summary, because county attractiveness is related to graduates’ motivation to seek jobs, it is applied in this study to explain the employment and entrepreneurship decisions of students.

### Family Factors

Family factors include parental support, parental education, and whether a person is the only child (Kracke, 1997). Family members, especially parents and guardians, play a pivotal role in the development of children’s occupational aspirations and career goals. Without parental approval or support, students and young people are often reluctant to pursue or even explore career possibilities of their choice. Although parents acknowledge their role and work hard to support their children’s professional development, parents do not want their children to make the same mistakes that they did. Moreover, parents are influential figures that intentionally or unintentionally expose children to job options and opportunities, as well as express potential expectations (Taylor et al., 2004). Parents’ expectations regarding their children’s future careers are determined by numerous complex factors, including their work conditions, their idea of a great career, and their estimate of their children’s actual capacity. Parents in the middle and working classes often wish for their children to land a higher-ranking job, frequently talk about future careers, and motivate them to perform well in school (Irwin and Elley, 2013).

According to Bryant et al. (2006), parents are critical role models in the career development process of the youth, as well as the main source of their occupational knowledge and beliefs. Kracke (1997) indicated that parental authority, openness to youth problems, and attention to career exploration are significantly correlated with children’s career exploration and have no significant correlation with parents’ educational background or the gender of the child. The effect of parental behavior on youth career exploration is characterized by a child-centered parent-child relationship, where the stronger the parental support and interaction, the more active the children are in career exploration. This relationship is independent of parents’ educational backgrounds or the gender of the child. Lindstrom et al. (2007) reported that family structure is not directly related to employment results, but family socioeconomic status is related to initial occupational decision-making and the development of a professional identity. For children who come from a family with lower socioeconomic status, their upbringing does not seem to limit their professional achievements but instead enhances their professional identity and vocational maturity. Moreover, career development is positively correlated with family support and the advocacy for activities that boost occupational intention. Keller and Whiston (2008) conducted research analyzing specific parental behaviors and youth career development. After controlling for Students’ grade level and gender, parental behavior was correlated with middle school Students’ career development. The degree to which students trust their career decision-making capabilities depends only on their perceived level of trust in their parents. Their mother’s occupation, parents’ work values, parents’ expectations, and parents’ communication with them were not significantly correlated with their vocational maturity. Compared with parental behavior, parental support could more robustly explain youth variations in professional performance. Generally, expressing interest in and support for students, especially regarding their career problems and plans, is more helpful to their career development than providing clear information about any particular career. Jacobs et al. (2006) concluded that parents’ occupational gender-role expectations for their children’s careers were closely related to their children’s future career expectations. From childhood throughout adolescence, parents’ occupational gender-role expectations regarding career and job choices align with their children’s occupational expectations 2 years later. This indicates that parental expectations regarding their children’s careers are related to their children’s future career expectations and choices. Career exploration requires a person to actively seek information from various professionals, such as consultants, which can deter students from making independent career choices and be a source of stress. Therefore, perceived parental security is crucial in that it can help students feel emotionally supported and protected. Olaosebikan and Olusakin (2014) reported that not only do parents negatively affect Students’ career choices, but parents’ influence on their career choices also is significant, and parents’ occupational attitudes significantly affect Students’ school choices as well. In addition, many previous studies have identified a positive correlation between family factors and entrepreneurship intention (Mustapha and Selvaraju, 2015; Hutasuhut, 2018; Nurmaliza et al., 2018). Ahmed et al. (2020) discovered that if an individual’s family does not support their entrepreneurial behavior, the entrepreneur’s enthusiasm is reduced, which in turn affects their future entrepreneurial behavior. In short, family dynamics, a crucial factor affecting employment, was applied in this study to explain the employment and entrepreneurial choices of college graduates.

## Methodology

### Research Model

**FIGURE 1:**
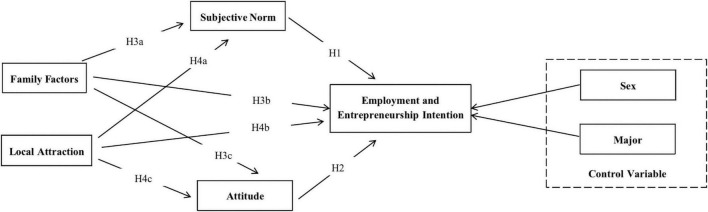
Research model.

The TRA is a widely applied theoretical framework proposed by Ajzen and Fishbein (1975). The basic assumption of TRA is that people are rational and, before displaying certain behaviors, they consider information when evaluating the meaning and consequences of the behavior. The theoretical model of this study is based on the existing literature. The study hypotheses are presented in Figure 1.

### Hypotheses

#### Theory of Reasoned Action

TRA, as a social psychological framework, has been recommended for research on variables that influence career choices (Cohen and Hanno, 1993). Personal intention, one of the core elements of TRA, produces the most accurate predictions for certain behaviors (e.g., staying local for work; Ajzen and Fishbein, 1975; Fishbein et al., 1980). The intention is jointly shaped by subjective norms and attitudes concerning occupations. Most studies have indicated that attitudes affect job-searching intentions (Van Hooft et al., 2004, 2005, van Hoye et al., 2009; Song et al., 2006; Van Hooft and De Jong, 2009; Zikic and Saks, 2009). Subjective norms reliably predict occupational intentions (Huang, 2011). Accordingly, this study proposes H1 and H2 based on TRA.

H1: A significant relationship exists between subjective norms and occupational and entrepreneurial intention.

H2: A significant relationship exists between occupational attitude and occupational and entrepreneurial intention.

#### Family Factors

Family factors affect occupational attitudes and behaviors, such as job satisfaction, relocation intention, and voluntary turnover (Blegen et al., 1988; Brett and Reilly, 1988). We deemed family factors, particularly parents, a crucial aspect of job searching. Parents are children’s primary companions in making decisions about future careers (Tynkkynen et al., 2010). Students talk about occupational issues most frequently with their parents (Otto, 2000) and claim that their parents have the greatest influence on their career and curriculum decisions (Mortimer et al., 2002). The TRA is a powerful explanatory model for this phenomenon. The opinions of a Student’s parents, relatives, and friends are referred to as the opinions of significant others regarding employment and entrepreneurship. Parents also affect Students’ overall evaluations of their occupational behavior and intentions. Accordingly, we propose the following hypotheses:

H3a: A significant relationship exists between family factors and subjective norms.

H3b: A significant relationship exists between family factors and occupational intention.

H3c: A significant relationship exists between family factors and occupational attitude.

#### Local Attractions

Local attractiveness is defined as the ability of a region to attract and retain talent. Counties attract talent by providing convenient facilities (Gottlieb et al., 1995). The experiences of outstanding talent already working in a county may affect the subjective norms of college graduates. In addition, benefits provided by counties positively affect the significant others of students (e.g., their classmates), which in turn influences the subjective norms of the students. County attractiveness includes employment policies promulgated by the government and the employment conditions offered by local enterprises to graduates, both of which affect graduates’ overall evaluation of their occupational behavior and confidence in their career success. Accordingly, we propose the following hypotheses:

H4a: A significant relationship exists between local attractiveness and subjective norms.

H4b: A significant relationship exists between local attractiveness and occupational intention.

H4c: A significant relationship exists between local attractiveness and occupational attitude.

### Construct Operationalization

This study mainly explored the intentions of and the influencing factors for college students to get a job or start their own business in local areas from the perspective of county education. To ensure the rationality of the research question design, this study, based on the relevant theoretical basis, sorted out the relevant literature, and designed a questionnaire on college Students’ intention to get a job or start their own business in local areas and the influencing factors from the perspective of county education. In addition, three professors were invited to modify the content of the preliminary questionnaire to integrate the topic sentence and content appropriateness, and ensure the content validity of the questionnaire scale.

The questionnaire in this study was measured using a seven-point Likert scale. To ensure the quality of the question design, the original English scale was translated. To ensure the accuracy of the translation, the questions were translated into Chinese by three professors in information education, and then translated into English by a professionally trained Chinese-English translator. Since the questionnaire was distributed in China, the questions were translated into Chinese and adjusted according to the situation of this study, so that the respondents could easily understand and answer these questions. For family factors, 11 questions were adopted from Kracke (1997) and had a Cronbach’s alpha of 0.83. Three questions of Dean and Suhartanto (2019) were employed to measure local attraction and had a Cronbach’s alpha of 0.811. Personal attitudes were measured using the nine-item questionnaire of Rodriguez-Gutierrez et al. (2020), which had a Cronbach’s alpha of 0.841. Employment intention was assessed using the four-item questionnaire of Rodriguez-Gutierrez et al. (2020); the Cronbach’s alpha was 0.906. Finally, for subjective norms, four questions from Rodriguez-Gutierrez et al. (2020) were employed and had a Cronbach’s alpha of 0.846. All the research questions are presented in Table 1.

**TABLE 1:** The research question.

Construct	Operational definition	Item	Literature
Family factors	The interference and influence of parents on their children’s choice of occupation	1. Regarding career selection, when I hold an opinion different from that of my family, they listen and consider my thoughts.	Kracke, 1997
		2. My family is willing to accept my thoughts and suggestions.	
		3. My opinions often influence my family’s decisions.	
		4. Regarding entrepreneurship and employment, my family often seek my opinion.	
		5. Despite different opinions, if I can convince my family, they would grant their permission (in relation to entrepreneurship or selecting a location of employment, for example).	
		6. My family often communicate with me about my employment interests and plans.	
		7. My family often provide me with information of various job opportunities.	
		8. My family analyze the advantages and disadvantages of entrepreneurship and employment (such as entrepreneurship and employment in county-level cities).	
		9. Regarding entrepreneurship and employment, my family believe that I should consider both family and career development.	
		10. Regarding entrepreneurship and employment, my family believe that I should consider both salary and promotion opportunities.	
		11. My family encourage me to obtain employment information as much as possible.	
Local attraction	The social environment and policies that promote the willingness of college students to remain in a place for employment	1. The housing prices and cost of living in county-level cites are low.	Dean and Suhartanto, 2019
		2. The policies of county entrepreneurship and employment attract me.	
		3. Entrepreneurship and employment in counties equate to less pressure at work.	
		4. Entrepreneurship and employment in counties are less competitive, facilitating job promotion.	
Employment intention	Intention of being employed or starting a new enterprise	1. I would choose the county-level city where my college is located or another county-level city for entrepreneurship and employment.	Rodriguez-Gutierrez et al., 2020
		2. I am currently preparing myself for work in county-level cities.	
		3. If I choose to work in county-level cities, I intend to work diligently.	
		4. In the future, I will likely undertake entrepreneurship and employment in county-level cities.	
Attitude	The positive or negative feelings an individual holds toward a behavior	1. I enjoy challenging myself.	Rodriguez-Gutierrez et al., 2020
		2. I enjoy dealing with challenges, and I can learn a lot from them.	
		3. I enjoy solving difficult tasks or problems.	
		4. To achieve my goals, I will work diligently.	
		5. Compared with simple tasks, I enjoy challenging myself and my abilities.	
		6. I enjoy challenging tasks, from which I can learn new skills.	
		7. When I choose a task I am interested in, I feel satisfied.	
		8. I enjoy tasks that require high levels of skill and talent.	
		9. I often gain knowledge and explore new personal skills.	
Subjective norms	Whether an individual perceives social pressure when adopting a specific behavior	1. My family supports my entrepreneurship and employment choices (such as remaining in a county-level city to work).	Rodriguez-Gutierrez et al., 2020
		2. My family thinks that remaining in a county-level city to work is a good choice.	
		3. My friends support my entrepreneurship and employment choices (such as remaining in a county-level city to work).	
		4. My friends are proud of me for my choices.	

### Data Collection

This study mainly explored the intentions of and the influencing factors for college students to get a job or start their own business in local areas, from the perspective of county education. College graduates are an important part of the talent needed for social development. The employment of college graduates in counties is not only related to the allocation of local talent resources but also plays an important role in the development of the county economy as well as cities. This study used both online and offline research methods. Online data collection was conducted mainly through Questionnaire Star^1^; the offline survey was conducted by issuing paper questionnaires to college students from a local university in Cixi City, Ningbo The survey was conducted from February to June 2021. To ensure the correctness of the survey and the reliability of the recovery, the questionnaires in this study were distributed by the researchers through the assistance of the professors in the university. Through the convenience sampling method, the questions were shared with all the students of the university and were answered voluntarily. Regarding questionnaire retrieval, we collected 833 questionnaires. Items were eliminated about the standards of Lin et al. (2021a). (1) Participants were estimated to require 5–10 min to complete the questionnaire. Responses from participants who spent less than 3 min completing the questionnaire were therefore considered invalid. (2) If all responses were identical (i.e., all numbers 1 or 7) within the same constructor had extreme values, they were considered invalid. (4) The questionnaire contains a reversed item to identify participants who failed to complete the questionnaire carefully. (5) Because the participants were soon-to-be graduates, we only retained responses from junior- and senior-year students. After abnormal samples were removed based on the aforementioned criteria, 798 (95.8%) valid questionnaires remained. The questionnaire content consisted of demographic information and items on subjective norms, local attraction, family factors, employment intention, and employment attitude.

With respect to demographic information, 326 of the participants were men, and 472 were women; 526 participants were junior-year students, and 272 were senior-year or above. As for the region in which they wish to seek employment, 69, 500, 162, 54, 0, and 12 reported their desire to remain in first-line cities (i.e., Beijing, Shanghai, Guangzhou, and Shenzhen), provincial capital cities or well-developed prefecture-level cities, regular prefecture-level cities, county or county-level cities, farming villages, and other settlement types, respectively. In terms of household registration type, there are 509 rural households and 289 urban households. There are 167 people in economics, 28 people in law, 37 people in education, 92 people in literature, 29 people in science, 169 people in engineering, 183 people in management, 86 people in art, and 7 people in other majors. The respondents tended to be women rather than men, because participation is voluntary, and most of them study business or education, with women in the majority. The research shows that the behavioral intention to use information system is affected by individual factors such as gender, age, and experience (Gefen et al., 2003). Therefore, the previous experience was applied to employment and entrepreneurship intentions, and college major and gender were used as control variables for the analysis of switching intention.

## Results

The statistical and analytical method employed in this study is the partial least squares structural equation modeling (PLS-SEM) technique based on SmartPLS 3.2.8, because it can visually check the relationship between the variables of interest, and the potential variables that are not observable and difficult to measure can also be obtained (Wong and Kwong, 2013). PLS-SEM has become a popular tool for analyzing such relationships (Sarstedt and Cheah, 2019). PLS-SEM outperforms CB-SEM in this study, especially when the purpose is exploratory research for theory formation; when the sample size is lower because the population is small; and when the distribution is non-normal (Gefen et al., 2011; Shiau, 2018; Hair et al., 2019; Khan et al., 2019; Shiau et al., 2019). For the reasons stated above, the PLS is a good SEM approach to use in the study. Two methods were selected to reduce common method variance at the questionnaire design and data collection phase. First, the questionnaire was processed in a paging way, so that the respondents could have an appropriate rest time, and the effect of time difference can reduce the influence of the same continuous scale on the variance of common methods (Podsakoff et al., 2003). Second, the existence of common method variance was confirmed using Harman’s single-factor test (Podsakoff et al., 2003). The factor explained shows that the value is 0.432, and the results exclude the potential threat of common method variance (Shiau and Luo, 2012). The variance inflation factor (VIF) is the ratio of variances when there is multicollinearity between variables and variances when there is no multicollinearity between variables. A larger VIF indicates more severe collinearity (Luo et al., 2020). The advantage of VIF lies in its simplicity: with the help of the stepwise VIF selection method, it is possible to reduce the risk of excluding correlated variables (Vörösmarty and Dobos, 2020).

According to Hair et al. (2017), the threshold of value tolerance is 0.10, and the VIF value is less than 5. All of these values were less than 2.395 (see Table 2), demonstrating that the study’s findings matched the criteria.

**TABLE 2:** Dimension reliability and validity.

Construct	Factor loading	Cronbach’s alpha	Composite reliability	AVE	VIF
Subjective norm	0.881*** 0.883*** 0.927*** 0.867***	0.912	0.938	0.792	2.284
Local attraction	0.873*** 0.906*** 0.938*** 0.934***	0.933	0.953	0.834	1.693
Family factor	0.875*** 0.861*** 0.858*** 0.875*** 0.814*** 0.873*** 0.843*** 0.864*** 0.796*** 0.827*** 0.817***	0.960	0.965	0.716	2.377
Attitude	0.921*** 0.930*** 0.916*** 0.871*** 0.882*** 0.921*** 0.807*** 0.868*** 0.886***	0.967	0.972	0.792	2.395
Employment and entrepreneurship Intention	0.883*** 0.926*** 0.864*** 0.938*** 0.864*** 0.938***	0.924	0.947	0.816	DV

^***^p < 0.05.

### Measurement Model

Henseler et al. (2014) explained that SRMR is considered a good matching value if the square root of the square difference of the sum of squares of the model implicit matrix and empirical correlation matrix is less than 0.10. In this study, we employed the goodness-of-fit measure because the SRMR composite factor model contains a formation structure. The SRMR for this study was 0.065, indicating an acceptable model.

The reliability and validity analysis of the measurement model was employed to evaluate the factor loading between the index constructs, Cronbach’s Alpha, combination reliability (CR), average variation extract (AVE), and discriminant validity. The reliability of each item was mainly detected and analyzed using factor loading and Cronbach’s alpha. According to the indicators in previous literature, Hair et al. (2017) suggested that both the factor loading and Cronbach’s alpha values should be above 0.7. Hair et al. (2006) advised that the result of combination reliability should be greater than 0.7, and the value of AVE should be greater than 0.5. All the statistical analysis results are presented in Table 2.

Discriminant validity tests the degree of discrimination between different constructs. The square root of AVE should be greater than the correlation coefficients between different constructs (Chin, 1998), so that the constructs have sufficient discriminant validity. Table 3 shows the correlation coefficient matrix among the various constructs. The diagonal line is the square root value of AVE, and the other values are the correlation coefficients between different constructs, to test the discrimination degree of the measured variables between different constructs. According to the results, the square root values of AVE for each construct were greater than the correlation coefficient between constructs, indicating that the results of each construct had discriminant validity. Table 4 shows the heterotrait-monotrait (HTMT) ratio. Henseler et al. (2015) stated that a HTMT ratio > 0.90 indicates unfavorable discriminative validity. Gold et al. (2001) and Teo et al. (2008) also adopted the HTMT < 0.90 standards. Clark and Watson (1995) and Kline (2011) applied a stricter standard that required a HTMT ratio < 0.85. In this study, all HTMT ratios were < 0.85, indicating that the study results fulfill even the strict requirement.

**TABLE 3:** Analysis of discriminant validity.

	Subjective norm	Local attraction	Family factor	Attitude	Employment and entrepreneurship intention
Subjective norm	**0.890**				
Local attraction	0.624	**0.913**			
Family factor	0.617	0.476	**0.846**		
Attitude	0.622	0.450	0.726	**0.890**	
Employment and entrepreneurship intention	0.697	0.669	0.403	0.447	**0.903**

*Bold values mean average variation extract square root.*

**TABLE 4:** Analysis of heterotrait–monotrait.

	Subjective norm	Local attraction	Family factors	Attitude	Employment and entrepreneurship intention
Subjective norm					
Local attraction	0.674				
Family factors	0.663	0.504			
Attitude	0.665	0.474	0.754		
Employment and entrepreneurship intention	0.754	0.719	0.428	0.473	

### Structural Model

**FIGURE 2:**
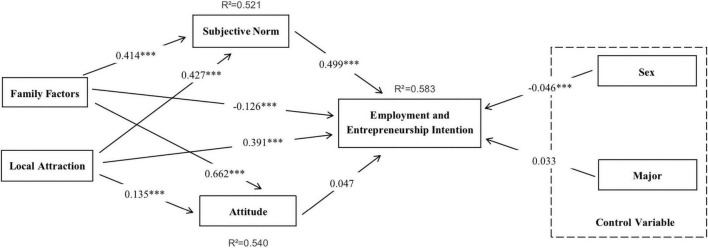
Structural model (*** significant at 0.05).

This study used bootstrap resampling method to evaluate PLS results with a sampling method of 5,000 samples (Hair et al., 2017). The analysis results of the structural model are shown in Figure 2. According to the analysis results, the overall explanatory power was 57.9%, the *R*^2^ of subjective norms was 51.9% and the *R*^2^ of attitudes was 54.5%. Thus, this study is predicted to be a model with good explanatory power.

In terms of the results of the H1 to H4c hypothesis, the statistical results of H1, H3a, H3b, H3c, H4a, H4b, and H4c were all supported. The subjective norm (H1) had a positive and significant impact on employment intention (*p* < 0.05). In addition, family factors had a significant impact on H3a and H3c at *p* < 0.05. Finally, the local attraction has a positive impact on subjective norms, employment intentions, and employment attitudes. Therefore, H4a, H4b, and H4c were supported.

The path coefficients in the model were compared using PLS-SEM to compare the two sets of samples. The significance of the difference in path coefficients was assessed according to the steps taken by Hair et al. (2006). One by one, the path coefficients were compared and validated. *P*-values were used to determine the significance of differences in path coefficients. The route coefficients of groups divided by sex and major were compared. Gender and major are two elements that influence behavior, according to existing research (Potard et al., 2017; Morales et al., 2018; Lin et al., 2021b). As a result, the main classification factors in this study were gender and major. In terms of sex (Table 5), there is a significant difference in the *p*-value of the subject norm on employment and entrepreneurial intention between the two groups, among which the male (0.016) is less than the female (0.071). At the same time, there is also a significant difference in the *p*-value of local attraction on attitude, among which the male (0.106) is greater than the female (0.001).

**TABLE 5:** Results of sex difference.

Relationship	Path coefficients (male)	*t*-value (male)	*p*-value (male)	Path coefficients (female)	*t*-value (female)	*p*-value (female)
Family factor - > Employment and entrepreneurship intention	0.594	7.293	0.000	0.443	7.651	0.000
Attitude - > Employment and entrepreneurship intention	0.454	6.293	0.000	0.410	5.786	0.000
Local attraction -> Attitude	0.086	1.618	0.106	0.158	3.442	0.001
Local attraction -> Employment and entrepreneurship intention	0.357	5.110	0.000	0.415	9.061	0.000
Family factor -> Subjective norms	0.404	5.669	0.000	0.421	6.600	0.000
Local attraction -> Subjective norms	0.723	15.886	0.000	0.625	14.896	0.000
Subjective norms -> Employment and entrepreneurship Intention	−0.157	2.409	0.016	−0.110	1.805	0.071
Family factor -> Attitude	0.036	0.578	0.563	0.054	0.866	0.387

## Conclusion

### Discussion

First, there is a significant correlation between subjective norms and employment intention (H1), which is similar to previous literature (Hooft et al., 2004). China is a country with collectivistic norms, which allows people to consider the opinions of people around them to a greater extent when choosing a job or starting their own business. Young people’s education and employment are often realized through families, which shows that students rely on their parents and friends and listen to their opinions when looking for jobs or starting their own business. However, there is no significant positive influence between employment or entrepreneurship attitude and employment or entrepreneurship intention (H2), which is contrary to the research report of Zikic and Saks (2009). This may be due to the difficult employment of Chinese college students, who pay more attention to the traditional objective conditions of the job, such as salary increases and difficulty of promotion (Huang et al., 2012). Students also give priority to job positions recommended by teachers, or workplaces where they have already worked as interns, recommended by their university (e.g., campus recruitment).

In addition, family factors have a significant impact on employment and entrepreneurship attitudes, subjective norms, and employment and entrepreneurship intention (H3c, H3a, H3b). This result is consistent with the conclusions of previous studies (Jacobs et al., 2006; Oren et al., 2013). It is apparent that college students pay attention to the opinions of their parents, relatives, or important people when choosing jobs or carving a career, and their suggestions can indirectly change Students’ employment and entrepreneurship intentions. Traditionally, Chinese people value “filial piety,” “carrying on the family line,” and “raising children for old age,” resulting in particularly strong family attachments. As a result, young people are often reluctant to pursue or even explore different careers without parental approval or support. Whether intentionally or unintentionally, children can understand and be exposed to specific occupations and implicit expectations from their parents (Taylor et al., 2004). Rural students are also greatly influenced by family factors in their employment and entrepreneurship preparation, and they are more likely to listen to their parents’ opinions than their urban counterparts. Therefore, guidance from parents or relatives plays a vital role in college Students’ employment and entrepreneurship.

Third, the results of this study show that local attraction has a significant impact on subjective norms, employment and entrepreneurship intentions, and employment attitudes (H4a, H4b, H4c). Many individual needs affect Students’ motivation for employment and entrepreneurship, and employment areas can meet these needs to a certain extent (Meng et al., 2008). College Students’ families or people around them may think that good conditions in the county support students in finding a job or starting their own business in the local area. An attractive county can encourage college students to find jobs or start their businesses in the local area.

Finally, there is a significant relationship between gender and employment and entrepreneurship intention. Redmond and McGuinness (2020) studied that, in general, women’s job satisfaction is higher than men’s. On average, women were more satisfied than men, and the gap persisted even after accounting for various personal, work, and family characteristics. In this study, for men, county attractiveness has no significant effect on employment attitude, but for women, it has a significant effect, which may be because women pay more attention to the working environment and conditions. But in the family factors and employment intention of significant sexual relations, on the other hand, for women, family factors for employment no significant sex, and for men, the family factor has a significant effect on employment intention, we suspect this may be due to a new era of economic development for the younger generation of women no longer like the past struggle with family, Men, on the other hand, hold themselves to a standard of excellence as “family-oriented.”

### Practical and Research Implications

This study explores the intentions of and influencing factors for college students to get a job or start their own business in local areas from the perspective of county education. College graduates are an important part of the talent needed for social development. The employment of college graduates in counties is not only related to the allocation of local talent resources but also plays an important role in the development of the county economy and the progress of cities.

From a theoretical perspective, family factors, employment and entrepreneurship attitudes, and subjective norms (Jacobs et al., 2006; Su et al., 2021) all have a significant impact on the employment and entrepreneurial inclinations of college students, proving that local attraction has a significant impact on the subjective norms of college Students’ employment and entrepreneurship. However, in past studies, only a few models have combined family factors and local attraction with the rational behavior theory, to study the employment and entrepreneurship of county college students. This study expanded these two new perspectives in rational behavior theory, developed a new framework for discussing county employment and entrepreneurship, and bridged a gap in the relevant literature. Although research paradigms and research models in the field of social psychology can be adopted as a reference, they are not enough to explain college graduates’ intentions to stay in a local county. While some scholars have paid attention to the employment flow of college graduates, most studies focus on the regional flow of college graduates nationwide from a national perspective, but lack focus on counties. This study takes independent colleges running at the county level as the research subject, and conducts a theoretical review and in-depth study on the employment of graduates in the county to further enrich the relevant theories and research results studies about graduates staying in colleges and universities.

Finally, Keller and Whiston (2008) argued that parents can greatly influence their children’s professional beliefs, values and goals. Parents’ influence on their children’s employment starts as early as the children’ admission to the school. In addition, we found in the process of research that, many parents hoped for their children to work or set up a business in the place where their families are located.

### Limitations and Future Research

Based on the data of a local university in Zhejiang Province, this study investigates and analyses the intention of and influencing factors for college students to find a job or start their own business in local counties, through a questionnaire survey. Although some conclusions have been drawn, the study has the following limitations. First, owing to human, financial, and time constraints, the sample size was small. This study only focused on the undergraduates of one county university and did not study all counties in Zhejiang Province, which limited the sample representativeness and might have affected the research results to some extent. However, the results can be employed to analyze the influence of different countries on the attractiveness of college graduates in the future. Second, this study only analyses local attractions and family factors, which is not enough to affect college Students’ intention to find jobs or start their own businesses. Factors such as school education and social concepts can be added in future research to further understand the influence of different factors. Third, this study did not focus on Students’ majors. According to previous studies (Su and Wu, 2021; Su et al., 2021), students of different majors may have different opinions on employment and entrepreneurship. For example, Joshi and Kuhn (2011) found that students who were not majoring in information systems had less social support than those who majored in information systems. Finally, as mentioned above, the author himself is a student of management, so most of the research subjects are students of management and economics, and there are a large number of female students in these two major categories, which inevitably leads to the majority of female students. In addition, most of the engineering subjects were contacted by the author with the help of the tutor. Due to the above situation, most of the research objects in this study are girls and the proportion of majors is not balanced, which leads to some limitations in this study.

## Data Availability Statement

The original contributions presented in the study are included in the article/supplementary material, further inquiries can be directed to the corresponding author/s.

## Ethics Statement

Ethical review and approval was not required for the study on human participants in accordance with the local legislation and institutional requirements. Written informed consent for participation was not required for this study in accordance with the national legislation and the institutional requirements.

## Author Contributions

All authors listed have made a substantial, direct, and intellectual contribution to the work, and approved it for publication.

## Conflict of Interest

The authors declare that the research was conducted in the absence of any commercial or financial relationships that could be construed as a potential conflict of interest.

## Publisher’s Note

All claims expressed in this article are solely those of the authors and do not necessarily represent those of their affiliated organizations, or those of the publisher, the editors and the reviewers. Any product that may be evaluated in this article, or claim that may be made by its manufacturer, is not guaranteed or endorsed by the publisher.

## Footnotes

1
https://www.wjx.cn/
